# E3 ubiquitin ligase BCA2 promotes breast cancer stemness by up-regulation of SOX9 by LPS

**DOI:** 10.7150/ijbs.92338

**Published:** 2024-04-29

**Authors:** Min Zheng, Wenjing Liu, Rou Zhang, Dewei Jiang, Yujie Shi, Yingying Wu, Fei Ge, Ceshi Chen

**Affiliations:** 1 Key Laboratory of Animal Models and Human Disease Mechanisms of the Chinese Academy of Sciences, Kunming Institute of Zoology, Chinese Academy of Sciences, Kunming, 650201, China.; 2 Kunming College of Life Sciences, University of Chinese Academy of Sciences, Kunming, Yunnan, China.; 3 The Third Affiliated Hospital, Kunming Medical University, Kunming, 650118, China.; 4 Academy of Biomedical Engineering, Kunming Medical University, Kunming, 650500, China.; 5 The First Affiliated Hospital, Kunming Medical University, Kunming, 650032, China.; 6 Department of Pathology, Henan Provincial People's Hospital, Zhengzhou University, Zhengzhou, China.

**Keywords:** BCA2, LPS, MyD88, SOX9, Breast cancer stem cells

## Abstract

Triple-negative breast cancer (TNBC) is the most malignant subtype of breast cancer. Breast cancer stem cells (BCSCs) are believed to play a crucial role in the carcinogenesis, therapy resistance, and metastasis of TNBC. It is well known that inflammation promotes stemness. Several studies have identified breast cancer-associated gene 2 (BCA2) as a potential risk factor for breast cancer incidence and prognosis. However, whether and how BCA2 promotes BCSCs has not been elucidated. Here, we demonstrated that BCA2 specifically promotes lipopolysaccharide (LPS)-induced BCSCs through LPS induced SOX9 expression. BCA2 enhances the interaction between myeloid differentiation primary response protein 88 (MyD88) and Toll-like receptor 4 (TLR4) and inhibits the interaction of MyD88 with deubiquitinase OTUD4 in the LPS-mediated NF-κB signaling pathway. And SOX9, an NF-κB target gene, mediates BCA2's pro-stemness function in TNBC. Our findings provide new insights into the molecular mechanisms by which BCA2 promotes breast cancer and potential therapeutic targets for the treatment of breast cancer.

## Introduction

Breast cancer is the most prevalent malignancy among women worldwide, exhibiting the highest incidence rate 1. Approximately 15-20% of breast cancers are triple-negative, characterized by negative expression of ERα (estrogen receptor), PR (progestern receptor) and HER2 (human epidermal growth factor receptor 2). Triple-negative breast cancer (TNBC) has a significantly higher risk of recurrence and metastasis than other breast cancer subtypes, leading to poor long-term outcomes 2. Currently, the treatment of TNBC is dominated by chemotherapy, although the emergence of PARP inhibitors, immunotherapies and the anti-Trop-2 antibody‒drug conjugate sacituzumab govitecan has made precision first-line therapy for TNBC possible 3. Consequently, there is an urgent need for innovative approaches to improve the prognosis of TNBC.

Cancer stem cells (CSCs) represent a distinct subset of cells with the capacity for tumor initiation and formation, long-term self-renewal, and the ability to differentiate into non-self-renewing cells 4. In breast cancer, it is common to use CD44 and ALDH1 expression to confirm the presence of BCSCs. CD44^high^/CD24^low^ and ALDH1-positive BCSC markers are more enriched in TNBC tumor tissues than in luminal A, luminal B, and HER2-positive subtypes 5, 6. Therefore, BCSCs have emerged as biomarkers and therapeutic targets for TNBC. Abnormalities in the activation or mutation of stem cell-associated genes are often reported to be associated with cancer aggressiveness and recurrence. The regulation of BCSCs self-renewal involves several major signaling pathways, including the Notch, Wnt, Hedgehog, and NF-κB signaling pathways. Targeting BCSCs holds significance for the treatment of TNBC.

Studies on the microbiome of tumors have revealed that various tumor types are composed of distinct microbiomes. The microbiome is primarily located within tumor and immune cells. An investigation into the microbiome of seven types of cancer discovered that breast cancer has the most diverse and rich microbiome. Such bacteria could then be a lipopolysaccharide (LPS) source which could trigger cancer progression 7. An increasing number of studies have shown that LPS is related to the development and metastasis of tumors, inducing the expression of cancer stem cell markers and promoting cancer stemness 8. In the tumor microenvironment, a variety of damage-associated molecular patterns (DAMPs) can bind to TLRs and induce the development of tumor drug resistance and metastasis. For example, the DAMP proteins HMGB1, S100, HSPs, API5 and RPS3 bind to TLR4 and accelerate tumor progression and metastasis 9. Previous studies have reported that LPS binds to TLR4 and leads to the activation of the downstream NF-κB pathway 10. In breast cancer, the expression of the TLR4 protein is linked to reduced survival rates 11. Furthermore, TLR4 supports the metastasis of breast cancer 12. TLR4 has also been identified as a marker of CSCs, promoting cancer progression and metastasis in hepatocellular carcinoma 8. Additionally, basal-like subtypes of breast cancer, particularly TNBC, have more prevalent NF-κB signaling 13. Accumulated evidence also suggests that NF-κB upregulates the expression of CD44^14^, ^15^. A study on liver regeneration after hepatectomy demonstrated that LPS stimulation increases the expression of stemness markers such as SRY-related high-mobility group Box 9 (SOX9) 16. It has also been shown that p65 acts as a transcription factor inducing the expression of SOX9^17^.

Breast cancer-associated gene 2 (BCA2), also referred to as RING finger protein 115 (RNF115), is an E3 ligase that belongs to the RING finger family. BCA2 has been found to be overexpressed in breast cancer, and its involvement has been observed in various physiological and pathological processes, including tumor formation, membrane receptor trafficking, DNA damage response and repair, innate immune signaling, and autophagosome formation^18^-^22^. BCA2 has been reported to catalyze the SUMOylation of IκBα in the presence of Ubc9, leading to stabilization of IκBα and inactivation of NF-κB. Moreover, BCA2 effectively suppressed HIV-1 transcription by inhibiting the NF-κB pathway^23^. In addition, BCA2 can interact with TLRs and play a role in the inhibition of the translocation of TLR2, TLR4, and TLR9 to the post-Golgi apparatus^24^.

In this study, we first demonstrated that BCA2 increases the proportion of BCSCs in TNBC. Furthermore, BCA2 positively regulates LPS-induced NF-κB signaling activation by augmenting the interaction between MyD88 and TLR4 while hindering the interaction between MyD88 and OTUD4. Moreover, we showed that BCA2 promotes stemness by increasing LPS-induced SOX9 expression. These findings suggest that BCA2 is a potent therapeutic target in TNBC.

## Materials and Methods

### Cell lines and cell culture

We obtained the human breast cancer cell lines HCC1806 and HCC1937 from the American Type Culture Collection (ATCC). These cell lines were cultured in RPMI-1640 (Gibco, C11875500BT) supplemented with 5% fetal bovine serum (FBS) (ExCell Bio, FSD500). The HEK293T cell line was cultured in high-glucose Dulbecco's modified Eagle's medium (DMEM) (Gibco, C11995500BT) supplemented with 5% FBS (ExCell Bio, FSD500). All cell lines were maintained in a 5% CO_2_ incubator at 37 °C. We authenticated the cell lines by short tandem repeat (STR) assays prior to the start of the experiments.

### Reagents and antibodies

LPS (L4391-1 MG), IL-1β (SRP3083), and TNFα (SRP2102) were purchased from Sigma‒Aldrich (St. Louis, MO). Anti-IKKα/b (2682), anti-p-IKKα (2078), anti-IκBα (9242), anti-p-IκBα (9246), anti-p65 (8242), anti-A20 (5630), and anti-p-p65 (3033) antibodies were purchased from Cell Signaling Technology (Danvers, MA, USA). Anti-Tubulin (T5168) and anti-OTUD4 (HAP036623) were purchased from Sigma‒Aldrich. Anti-MyD88 (sc-74532), anti-TLR4 (sc-293072), and anti-IRAK-1 (sc-5288) antibodies were purchased from Santa Cruz Biotechnology (Santa Cruz, CA, USA). The anti-RNF115/BCA2 antibody (ab187642) was purchased from Abcam (Cambridge, MA). Cells were harvested, lysed in RIPA lysis buffer supplemented with 1×Proteinase Inhibitor Cocktail (MedChemExpress, Monmouth Junction, NJ, USA, HY-K0010) and quantified using a BCA kit (Thermo Fisher). Briefly, ~40 µg of each protein sample was subjected to SDS‒PAGE and transferred to polyvinylidene fluoride (PVDF) membranes (Merck Millipore, Germany, #IPFL00010). The membrane was blocked with 5% milk for one hour and then incubated with primary antibody overnight at 4 °C. The membrane was incubated with horseradish peroxidase (HRP)-conjugated secondary antibodies (Invitrogen, #31460 & #31430) for 1 hour at RT. Chemiluminescence was detected using an ImageQuant LAS4000 biomolecular imager (GE, USA) with Western HRP substrate (US Everbright, S6009 L).

### Flow cytometry

We performed ALDH assays using the ALDEFLUOR^TM^ Kit (Stem Cell Technologies, Vancouver, BC, Canada, #01700) according to standard protocols. Specifically, 50,000 cells were collected and resuspended in PBS buffer containing 2% FBS. Then, 5 µL of activated reagent was added. Half of the samples (0.5 ml) were added to a control tube containing 5 µL DEAB buffer. Both sets of samples were incubated at 37 ℃ for 40 minutes, followed by centrifugation at 500 × g for 5 minutes. Cells were resuspended in 400 µL of experimental buffer and analyzed by flow cytometry. The CD24/CD44 assay was performed with antibodies against CD24 (BD biosciences, CA, USA, #555428) and CD44 (BD biosciences, CA, USA, #555478) according to the manufacturer's manuals. Briefly, for CD24/CD44 staining, the cells were incubated with anti-CD24 and anti-CD44 antibodies on ice for 25 minutes after centrifuging at 500 × g for 5 minutes, the cells were collected, washed once using HF solution, and applied for flow cytometry analysis.

### Mammosphere culture

Mammosphere assays were performed using the Mammosphere Culture Kit (Stem Cell Technologies, #05620). For the culture of HCC1937, cells were removed from the incubator, washed with PBS, and digested into single cells by adding trypsin. The digestion was terminated by centrifuging the cells at 800 × g for 4 minutes and discarding the supernatant. The cells were resuspended and counted using a cell counter. The cells were resuspended and counted using a cell counter. A total of 1000 cells were added to each well of a 24-well ultralow adherence plate. Three replicate wells were set up for each group and incubated in the incubator; 100 µL of microspheres was added every 3 days; microsphere formation was observed during the incubation process and generally after 10-14 days of incubation; the microspheres formed were photographed, and the number of microspheres was counted and then statistically analyzed.

### Endogenous ubiquitination assays

Stable BCA2 knockout HCC1806 cells were seeded in 10 cm dishes, walled off and then treated with LPS (500 ng/mL) for 30 minutes. The cells were harvested in lysis buffer (50 mM Tris-Cl and 1% SDS; pH 6.8), and cell lysates containing 1 mL of lysis buffer per well were denatured at 95 °C for 15 minutes to remove proteins. BSA buffer (50 mM Tris-Cl, 180 mM NaCl, 0.5% NP-40 and 0.5% BSA; pH 6.8) was added to dilute the samples. Protein A/G beads (30 µL/sample; prewashed three times with BSA buffer) were added to the immunoprecipitates overnight in a cold room (4 °C) for spinning. These beads were washed three times with 1 mL of frozen BSA buffer, resuspended in 30 µL of 2× SDS‒PAGE sample buffer and boiled for 10 minutes.

### Immunoprecipitation

For exogenous immunoprecipitation, we cotransfected HEK293T cells with PCDH-Flag-MyD88 or PCDH-Flag-TLR4 with pcDNA3.1-BCA2 using PEI transfection reagent (Polysciences). After 48 hours, the cells were harvested and lysed with lysis buffer (50 mM Tris, 150 mM NaCl, 1 mM EDTA, 1% NP-40, 10% glycerol; pH 7.5). A protease inhibitor cocktail was added to the cell lysate. Cell lysates were incubated with anti-FLAG magnetic beads (MedChemExpress, Monmouth Junction, NJ, USA, HY-K0207) for 6 hours at 4 °C. The beads were washed and eluted, and the supernatant was analyzed by Western blotting. For endogenous immunoprecipitation, HCC1806 cells were lysed with lysate followed by mouse normal IgG; the lysate was spiked with MyD88 antibody (Santa, sc-74532,) or BCA2 antibody (Novus, NBP1-85586) and incubated overnight at 4 °C. Antibody-coupled lysates were incubated with Protein A/G beads for 2 hours. The beads were thoroughly washed and eluted, and the supernatants were analyzed by Western blotting as described above.

### GST pull-down assay

BCA2 and MyD88 cDNAs were cloned into the pEBG vector. The truncated forms of BCA2 and MyD88 were constructed by ligating cDNA fragments of different lengths to the pEBG vector. In HEK293T cells transfected with GST fusion proteins. Cell lysates were collected after 48 h. Cell lysates were incubated with glutathione-Sepharose 4B beads (GE Healthcare, Uppsala, Sweden) for 4 hours. Beads were washed 5 times with lysis buffer and 1× sodium dodecyl sulfate (SDS) sample buffer was boiled for 10 minutes and then subjected to Western blotting assay.

### Transfection and production of lentiviral particles

For the construction of stably overexpressing BCA2 cells, HEK293T cells were transfected with PCDH-Flag-BCA2, PCDH-Flag-BCA2(C228/231A) or empty, and the packaging plasmids used were PMDL, VSVG and REV. Lentiviruses were harvested after 48 hours and then stored at -80 °C. Lentiviruses were added to HCC1806 and HCC1937 cells along with 8 μg/mL polypyrene (Sigma, H9268), and after 24 hours, the medium was replaced with fresh medium supplemented with 1 μg/mL puromycin (InvivoGen) to select for stably infected cells.

### Generation of BCA2 KO cell lines

The sgRNAs targeting BCA2 were constructed using plasmid Lenti-CRISPRv2 and transferred via lentivirus into HCC1806 and HCC1937 cells. The knockout effect was evaluated by Western blotting. The sgRNA sequences used in this study are as following: BCA2 sgRNA#1: 5'-CACCGTTCGGCGGCCGGGGCGGACT-3', BCA2 sgRNA#2: 5'- CACCGAAACCGGTGGGCGGCTACAG-3', BCA2 sgRNA#5: 5'-CACCGTCCGCCGCGGCCGTCCGAGA-3'.

### *In vivo* limited dilution assay

HCC1806 cells (1×10^4^, 5×10^3^, 2×10^3^) were suspended in 75 µL of Matrigel (Corning, BD Biocoat, #354234) and phosphate-buffered saline (PBS) at a 1:1 ratio and injected into the fat pads of 4- to 5-week-old BALB/C nude mice obtained from Hunan SJA Laboratory Animal Co. Ltd. (Changsha, Hunan, China). Tumor size was monitored weekly after injection, and mice were sacrificed when the tumor diameter reached 1.0-1.5 cm. Tumor volume was calculated as length × width^2^/2. This animal experimentation was approved by the animal ethics committee of Kunming Medical University (KMMU2020132).

### Statistical Analysis

Unless otherwise specified, we repeated all experiments at three times. Data are expressed as the mean ± SD. Statistical analyses were performed using Student's t test, unless otherwise noted. GraphPad Prism 8 (GraphPad Software Inc. La Jolla, CA, USA) was used for all statistical analyses. P values less than 0.05 were considered significant, **, P < 0.01, *, P < 0.05, n.s., not significant, t, test.

## Results

### BCA2 knockout inhibits the proportion of BCSCs in TNBC cells

**Figure 1:**
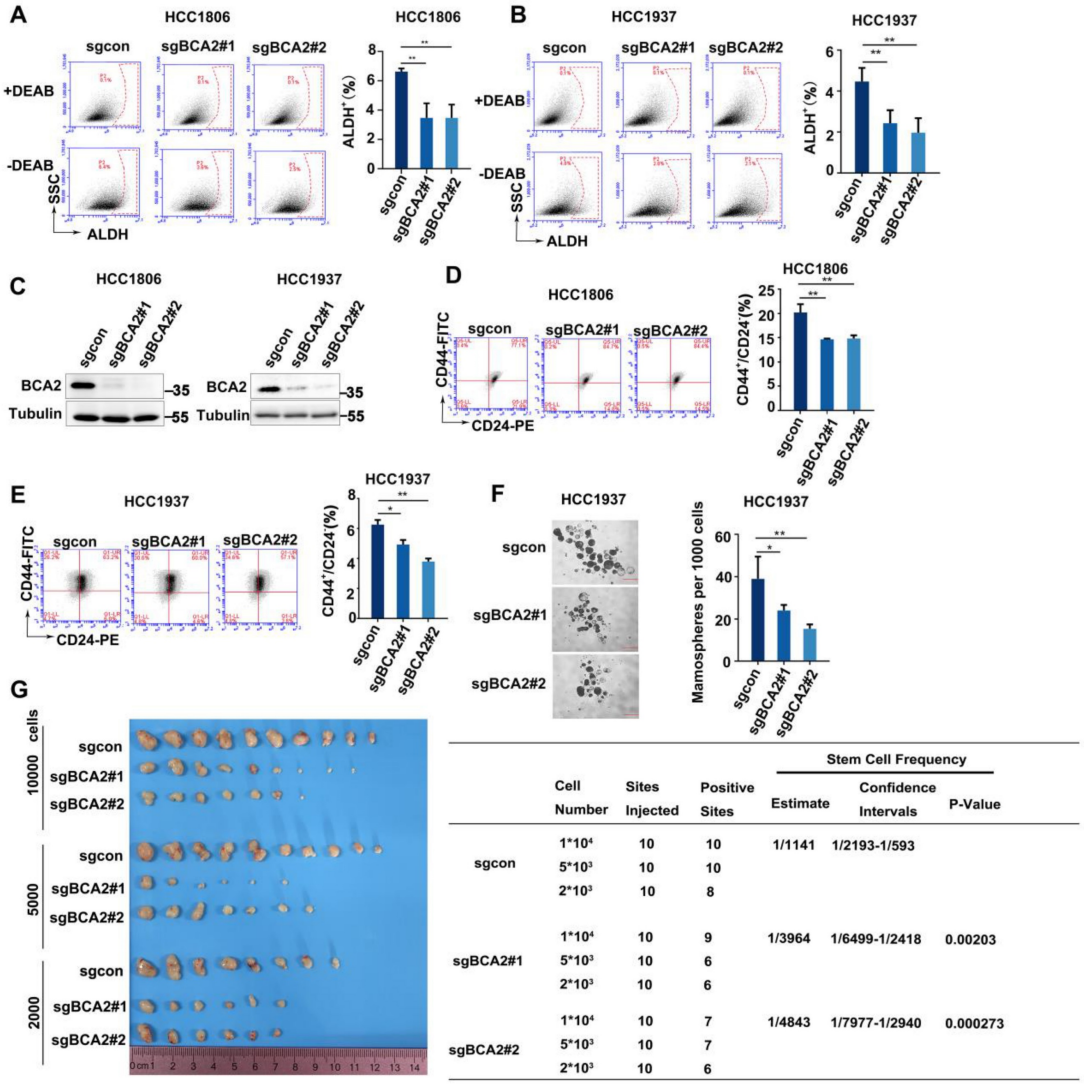
BCA2 knockout results in a decreased proportion of BCSCs. **(A)** ALDH activity detected by ALDEFLUOR assay in HCC1806 cells with BCA2 knockout. Statistical results of the ALDH+ BCSC population in HCC1806 cells. **(B)** ALDH activity detected by ALDEFLUOR assay in HCC1937 cells with BCA2 knockout. Statistical results of the ALDH+ BCSC population in HCC1937 cells. **(C)** Protein expression levels of BCA2 in HCC1806 and HCC1937 cells with BCA2 knockout or their mock cells.** (D)** CD44^+^/CD24^-^ BCSC population analysis in HCC1806 cells with BCA2 knockout. Statistical results of the CD44^+^/CD24^-^ BCSC population in HCC1806 cells.** (E)** CD44^+^/CD24^-^ BCSC population analysis in HCC1937 cells with BCA2 knockout. Statistical results of the CD44^+^/CD24^-^ BCSC population in HCC1937 cells.** (F)** Mammosphere formation assay in HCC1937 cells with BCA2 knockout. Scale bar =250µM. Statistical results of the mamosphere formation ratio in HCC1937 cells.** (G)** The limited dilution assay was used to calculate the stem cell frequency in HCC1806 xenograft tumors. Statistical results of the tumorgenesis rate in HCC1806 cells. *, *p* < 0.05 **,* p* < 0.01

The expression of BCA2 is increased in both breast cancer cells and tissues 25. However, the exact role of BCA2 in breast cancer remains unclear. We previously reported that BCA2 promotes breast cancer cell proliferation by targeting p21 for ubiquitin-mediated degradation 19. To evaluate the impact of BCA2 on BCSCs, we first examined the proportion of ALDH+ cells after depleting BCA2. Our findings revealed a significant reduction in the proportion of ALDH+ BCSCs in the HCC1806 and HCC1937 cells following BCA2 knockout (Fig. 1A-C). Consistent with these results, we also observed a notable reduction in the proportion of CD44^high^/CD24^low^ BCSCs in the HCC1806 and HCC1937 cells following BCA2 knockout (Fig. 1D, E). Furthermore, mammosphere formation assays performed on HCC1937 cells revealed that knockout of BCA2 markedly impaired the capacity of BCSCs to form mammospheres (Fig. 1F). HCC1806 could not form mammospheres well (data not shown), so we did not perform this experiment in HCC1806. Most importantly, limited dilution tumorigenesis assay revealed that BCA2 knockout in HCC1806 cells significantly reduced the proportion of BCSCs as well as their capacity to form tumors in nude mice (Fig. 1G). These data suggested that BCA2 promotes the proportion of BCSCs in TNBC.

### LPS increases the proportion of BCSCs via BCA2-mediated upregulation of SOX9 expression

**Figure 2:**
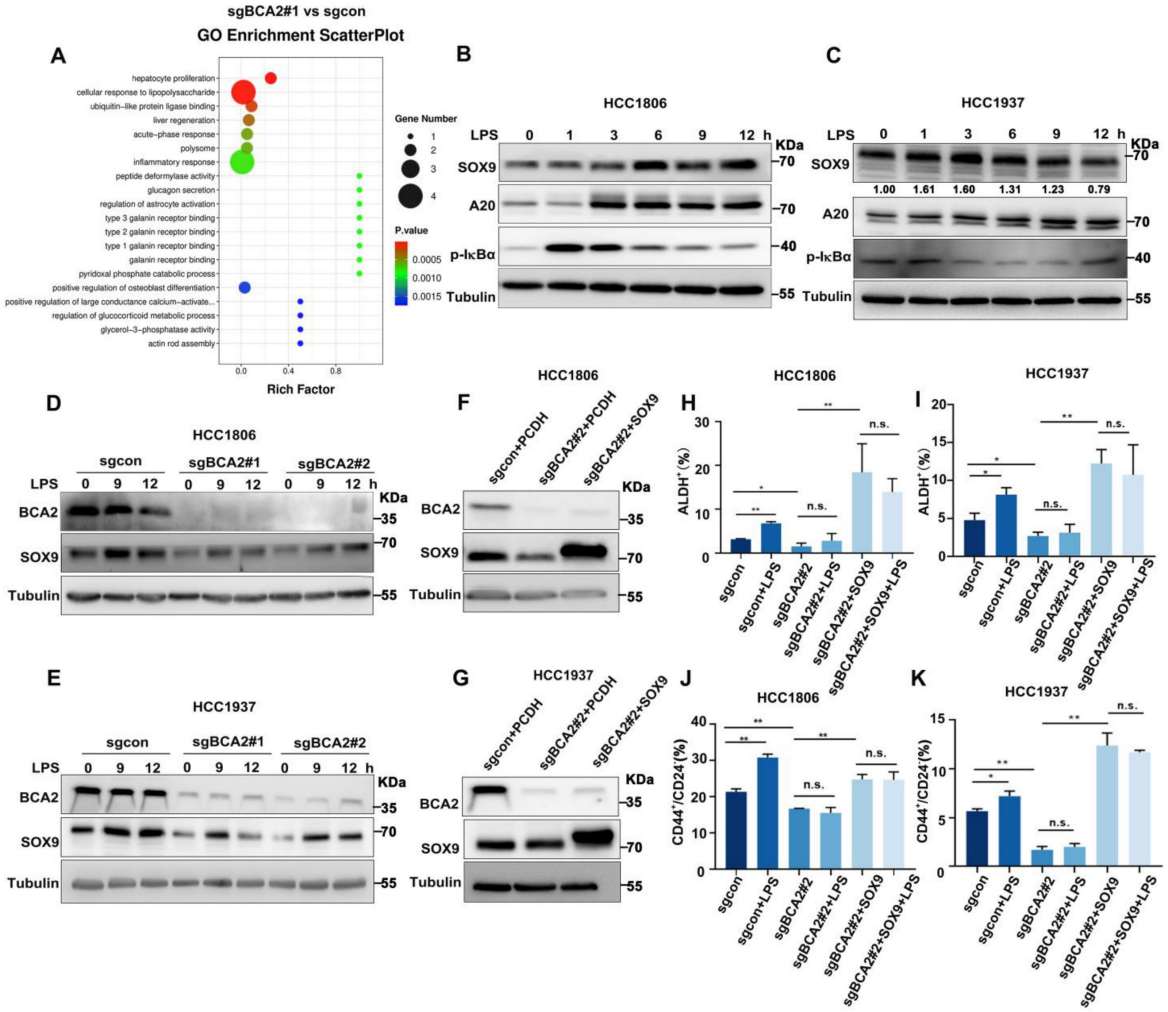
LPS promotes the proportion of BCSCs via BCA2-mediated upregulation of SOX9 expression. **(A)** GO showing enrichment of cellular response to LPS in BCA2 differentially expressed genes based on RNA-Seq data (sgBCA2#1 vs sgcon).** (B)** Immunoblot analysis of SOX9, A20, p-IκBα and tubulin in HCC1806 cells stimulated with LPS (50 ng/mL) for 0-12 hours.** (C)** Immunoblot analysis of SOX9, A20, p-IκBα and tubulin in HCC1937 cells stimulated with LPS (50 ng/mL) for 0-12 hours.** (D)** Immunoblot analysis of BCA2, SOX9 and tubulin in BCA2 knockout HCC1806 cells stimulated with LPS (50 ng/mL) for 0, 9, and 12 hours.** (E)** Immunoblot analysis of BCA2, SOX9 and tubulin in BCA2 knockout HCC1937 cells stimulated with LPS (50 ng/mL) for 0, 9, and 12 hours.** (F)** The knockout efficiency of BCA2 and overexpression of SOX9 at the protein level in HCC1806 cells.** (G)** The knockout efficiency of BCA2 and overexpression of SOX9 at the protein level in HCC1937 cells. **(H)** ALDH activity detected by ALDEFLUOR assay in HCC1806 cells with BCA2 knockout followed by stimulation with LPS (50 ng/mL) or BCA2 knockout followed by SOX9 rescue and stimulation with LPS (50 ng/mL). Statistical results of the ALDH+ BCSC population in HCC1806 cells (*, *p* < 0.05 **,* p* < 0.01, n.s. , not significant).** (I)** ALDH activity detected by ALDEFLUOR assay in HCC1937 cells with BCA2 knockout followed by stimulation with LPS (50 ng/mL) or BCA2 knockout followed by SOX9 rescue and stimulation with LPS (50 ng/mL). Statistical results of the ALDH+ BCSC population in HCC1937 cells (*, *p* < 0.05 **,* p* < 0.01, n.s. , not significant).** (J)** CD44^+^/CD24^-^ BCSC population analysis in HCC1806 cells with BCA2 knockout followed by stimulation with LPS (50 ng/mL) or BCA2 knockout followed by SOX9 rescue and stimulation with LPS (50 ng/mL). Statistical results of the CD44^+^/CD24^-^ BCSC population in HCC1806 cells (*, *p* < 0.05 **,* p* < 0.01, n.s. , not significant).** (K)** CD44^+^/CD24^-^ BCSC population analysis in HCC1937 cells with BCA2 knockout followed by stimulation with LPS (50 ng/mL) or BCA2 knockout followed by SOX9 rescue and stimulation with LPS (50 ng/mL). Statistical results of the CD44^+^/CD24^-^ BCSC population in HCC1937 cells (*, *p* < 0.05 **,* p* < 0.01, n.s. , not significant).

To characterize the mechanism by which BCA2 promotes the proportion of BCSCs in TNBC, we conducted RNA-seq analysis on HCC1806 cells with stable BCA2 knockout. GO enrichment analysis revealed that BCA2 knockout significantly affected the signaling pathways associated with cellular responses to LPS in HCC1806 cells (Fig. 2A and Fig. S2A). LPS stimulation has been reported to increase SOX9 expression, and p65 in the NF-κB pathway is a direct transcription factor that induces SOX9 expression 16, 17. We therefore speculated that BCA2 might regulate the expression of the stemness transcription factor SOX9. We then examined the expression of stemness-associated factors, including SOX9. In cells with stable knockout of BCA2, BCA2 knockout stably downregulated the expression level of SOX9 (Fig. S1A, B). Based on these findings, we stimulated HCC1806 and HCC1937 cells with LPS and observed that this induction resulted in the expression of A20, a target gene of NF-κB, and SOX9, a widely recognized stem cell transcription factor (Fig. 2B,C). As expected, LPS-induced SOX9 expression was significantly downregulated following BCA2 knockout (Fig. 2D,E). Thus, we proposed that BCA2 is needed for LPS to induce the expression of SOX9. To further confirm the role of BCA2 in promoting breast cancer stemness through SOX9, we generated HCC1806 and HCC1937 cells with BCA2 knockout and subsequently overexpressed SOX9 (Fig. 2F,G). Flow cytometry analysis demonstrated that LPS stimulation significantly increased the proportion of ALDH+ and CD44^high^/CD24^low^ BCSCs in the control group but not in the BCA2 knockout group, suggesting that BCA2 contributes to the promotion of the proportion of BCSCs by LPS (Fig. S2B,C). Furthermore, when SOX9 expression was restored following BCA2 knockout, the proportion of ALDH+ and CD44^high^/CD24^low^ BCSCs in HCC1806 and HCC1937 cells was significantly increased (Fig. 2H-K and Fig. S2D-G). These data suggested that BCA2 enhances the protein expression of SOX9, leading to an increase in the proportion of BCSCs in TNBC induced by LPS.

### BCA2 is essential for LPS-induced NF-κB signaling pathway activation independent of its E3 ligase activity

**Figure 3:**
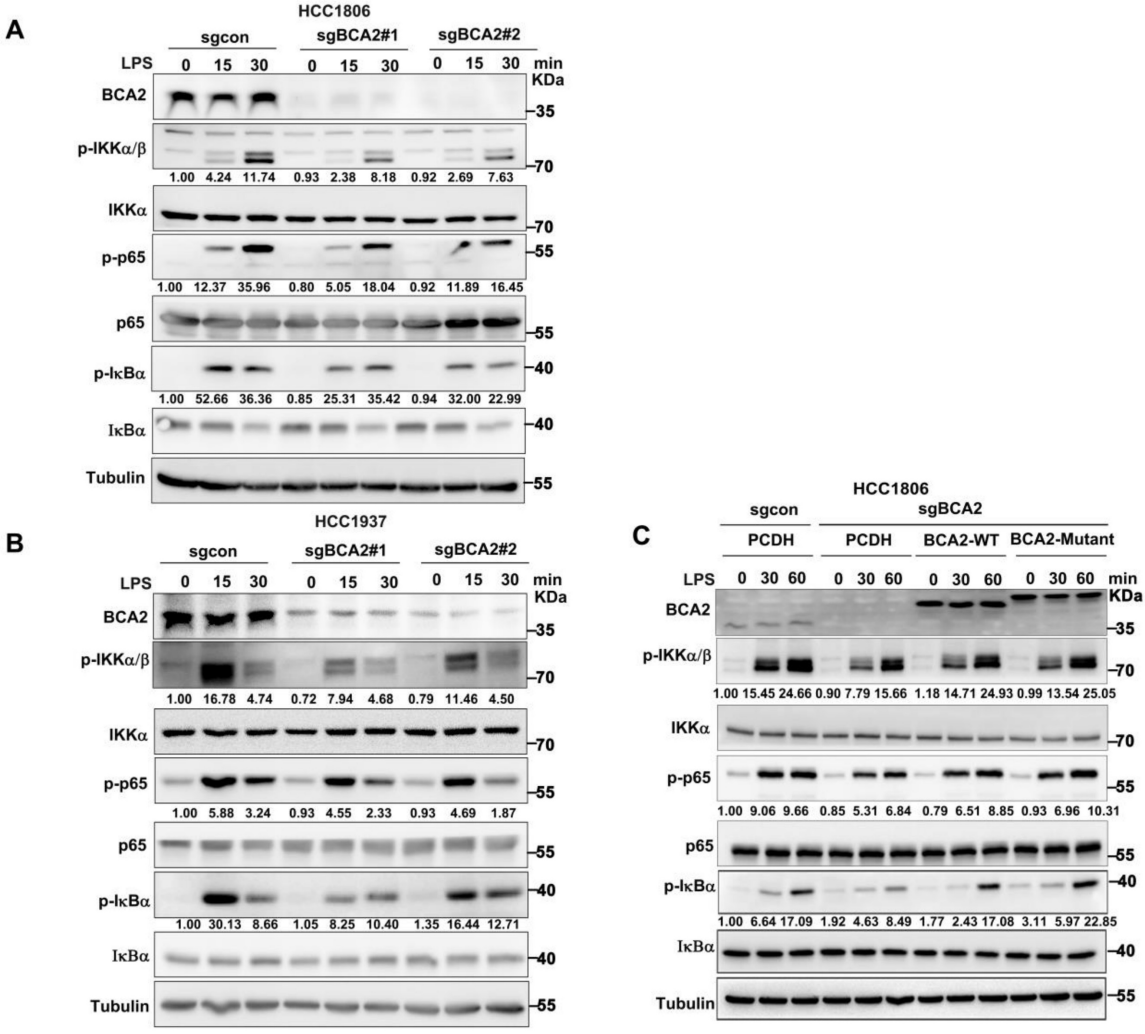
BCA2 enhances LPS-induced activation of the NF-κB pathway. **(A)** Immunoblot analysis of total and phosphorylated IKKα/β, p65, and IκBα in BCA2 knockout HCC1806 cells stimulated with LPS (500 ng/mL) for 0-30 minutes. **(B)** Immunoblot analysis of total and phosphorylated IKKα/β p65 and IκBα in BCA2 knockout HCC1937 cells stimulated with LPS (500 ng/mL) for 0-30 minutes. **(C)** Immunoblot analysis of total and phosphorylated IKKα/β, p65, and IκBα in BCA2 rescue after knockout of BCA2 in HCC1806 cells stimulated with LPS (500 ng/mL) for 0-60 minutes.

Given the ability of LPS to promote SOX9 expression by activating the NF-κB pathway, we hypothesised that BCA2 would promote LPS-induced SOX9 protein expression, which promotes the proportion of BCSCs. Therefore, we speculated that BCA2 is involved in LPS-induced NF-κB pathway activation. To investigate this, we knocked out BCA2 in HCC1806 and HCC1937 cells and stimulated the cells with LPS. We found that BCA2 knockout impeded the LPS-induced activation of the NF-κB pathway, as indicated by p-IKKα/β, p-p65 and p-IκBα (Fig. 3A,B). However, BCA2 knockout had no obvious effects on TNFα or IL-1β-induced activation of the NF-κB pathway. These results further confirmed the conclusion that BCA2 is required for LPS-induced but not TNFα or IL-1β-induced activation of the NF-κB pathway (Fig. S3A-D). We concluded that BCA2 specifically promotes the activation of the NF-κB pathway induced by LPS. Furthermore, we investigated whether the E3 ubiquitin ligase activity of BCA2 is needed for activation of the NF-κB signaling pathway. We restored wild-type (WT) and catalytically inactive BCA2(C228/231A) in BCA2 knockout HCC1806 cells and found that both BCA2 WT and the enzyme-dead mutant were able to support LPS-induced NF-κB signaling pathway activation (Fig. 3C). These data suggested that BCA2 is essential for LPS-induced NF-κB signaling pathway activation independent of its E3 ligase activity.

### BCA2 interacts with MyD88 and TLR4 and promotes their interaction

**Figure 4:**
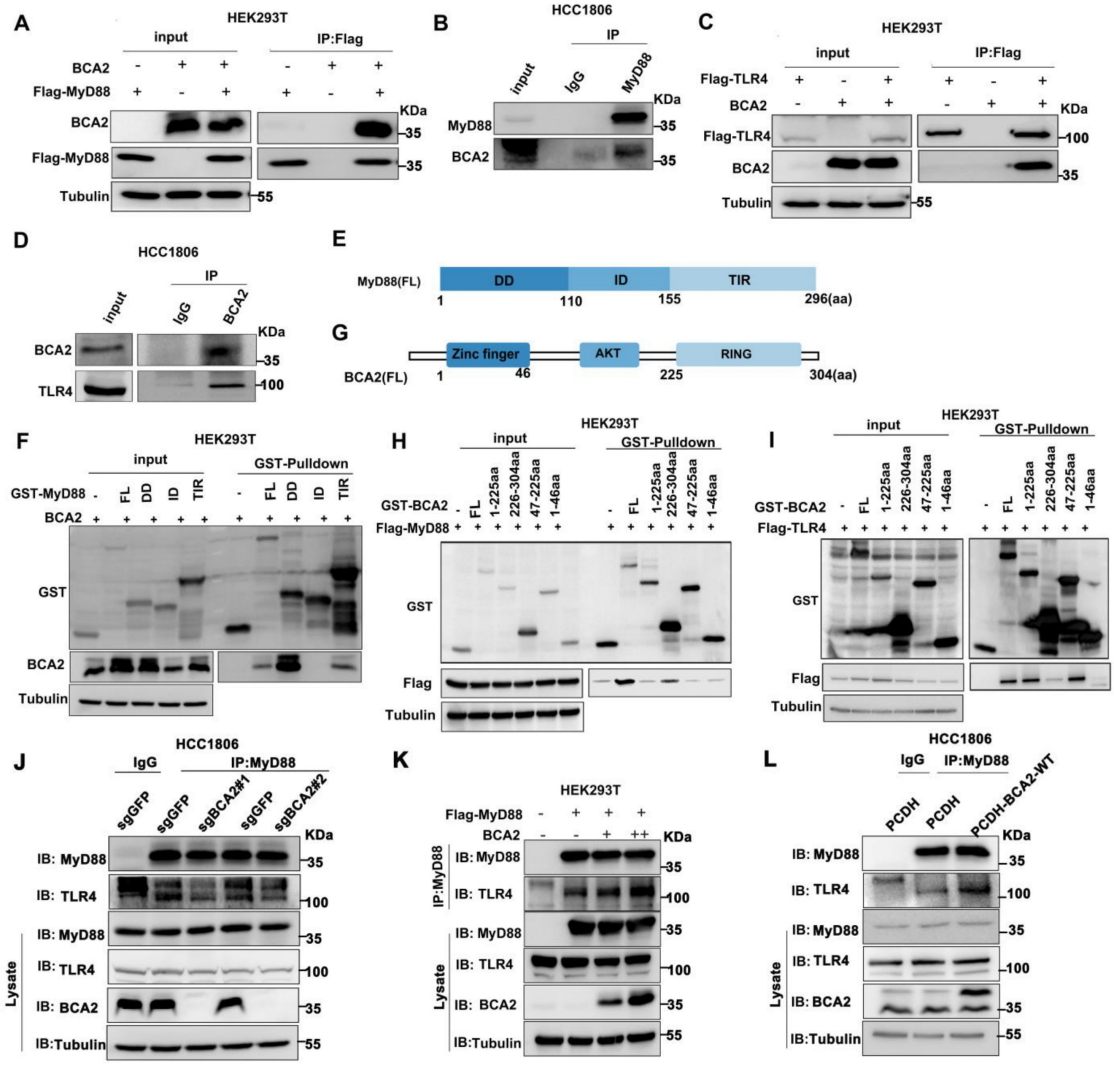
BCA2 interacts with the TLR4 and MyD88 proteins. **(A)** BCA2 was coimmunoprecipitated with Flag-MyD88 in HEK293T cells. **(B)** MyD88 was immunoprecipitated with endogenous BCA2 in HCC1806 cells. **(C)** BCA2 was coimmunoprecipitated with Flag-TLR4 in HEK293T cells. **(D)** TLR4 was immunoprecipitated with endogenous BCA2 in HCC1806 cells. **(E)** Diagrams of GST-fused MyD88 fragments. **(F)** HEK293T cells were transfected with different GST-fused MyD88 fragments and BCA2. After 48 hours of transfection, cells were harvested for GST pull-down. **(G)** Diagrams of GST-fused BCA2 fragments. **(H)** HEK293T cells were transfected with different GST-fused BCA2 fragments and Flag-MyD88. After 48 hours of transfection, the cells were harvested for GST pull-down. **(I)** HEK293T cells were transfected with different GST-fused BCA2 fragments and Flag-TLR4. After 48 hours of transfection, cells were harvested for GST pull-down. **(J)** In HCC1806 cells with BCA2 knockout, MyD88 was immunoprecipitated with endogenous TLR4. **(K)** HEK293T cells were transfected with the indicated plasmids, and then coimmunoprecipitation and immunoblot analysis were performed. **(L)** In HCC1806 cells overexpressing BCA2, MyD88 was immunoprecipitated with endogenous TLR4.

Because BCA2 responds only to the NF-κB signaling pathway activated by LPS but not to other NF-κB activitors such as TNFα and IL-1β, we speculate that BCA2 may regulate upstream signaling above IKK. It is well known that LPS binds to TLR4. TLR4 recruits the adaptor protein MyD88, which in turn recruits IRAK4 to form the Myddosome. IRAK4 is activated by trans-autophosphorylation, which then activates IRAK1/2 by phosphorylation, which in turn activates the downstream signaling pathway 26. We conducted immunoprecipitation (IP) experiments to examine the interaction among BCA2, TLR4, and MyD88. Indeed, we showed that BCA2 was coimmunoprecipitated by Flag-MyD88 (Fig. 4A). Similarly, endogenous MyD88 was found to interact with endogenous BCA2 in HCC1806 cells (Fig. 4B). Furthermore, we found that BCA2 was coimmunoprecipitated by Flag-TLR4 (Fig. 4C). The interaction between endogenous TLR4 and BCA2 was also detected in HCC1806 cells (Fig. 4D). We further mapped the specific domains involved in these interactions. BCA2 binds to the DD and TIR domains of MyD88 (Fig. 4E, F). Additionally, the RING domain of BCA2 interacts with MyD88, while the AKT domain of BCA2 is bound by TLR4 (Fig. 4G-I). Since the different regions of BCA2 bind to MyD88 and TLR4, we speculate that BCA2 promotes the NF-κB signaling pathway by facilitating TLR4 recruitment of MyD88. As expected, BCA2 knockout resulted in a decreased interaction between TLR4 and MyD88 (Fig. 4J). Consistently, overexpression of BCA2 enhanced the interaction between TLR4 and MyD88 (Fig. 4K, L). These data suggested that BCA2 facilitates the recruitment of MyD88 to TLR4.

### BCA2 inhibits the interaction between MyD88 and OTUD4 and increases MyD88 ubiquitination

**Figure 5:**
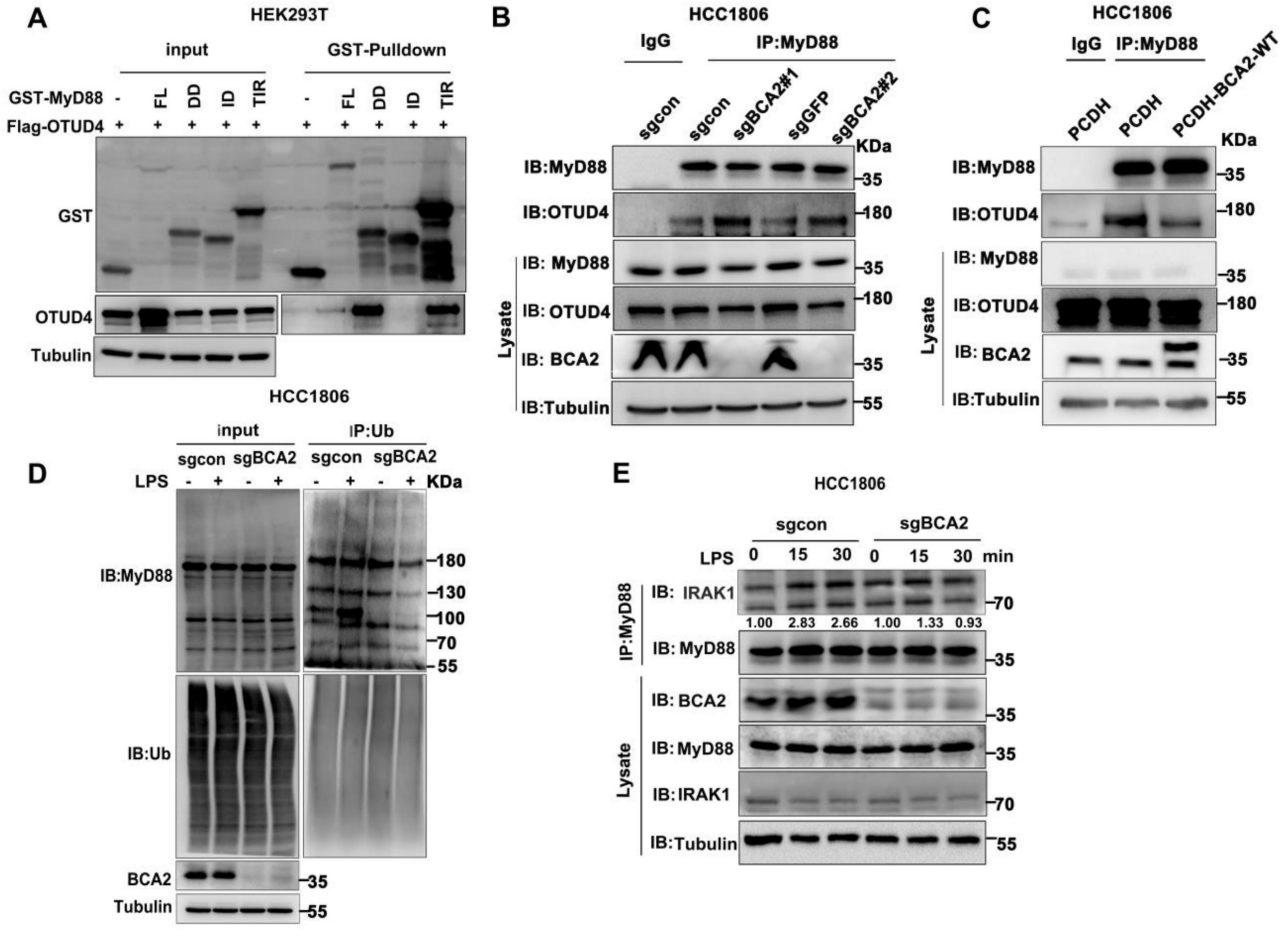
BCA2 facilitates MyD88 interaction with TLR4 and inhibits MyD88 interaction with OTUD4. **(A)** HEK293T cells were transfected with different GST-fused MyD88 fragments and Flag-OTUD4. After 48 hours of transfection, cells were harvested for GST pull-down. **(B)** In HCC1806 cells with BCA2 knockout, MyD88 was immunoprecipitated with endogenous OTUD4. **(C)** In HCC1806 cells overexpressing BCA2, MyD88 was immunoprecipitated with endogenous OTUD4. **(D)** Endogenous ubiquitination experiments were performed in HCC1806 cells with BCA2 knockout after stimulation with LPS (500 ng/mL) using a ubiquitin Ab. **(E)** HCC1806 cells with BCA2 knockout were stimulated with LPS (500 ng/ml) for 0-30 minutes before immunoprecipitation and immunoblot analysis.

OTUD4 has been reported to inhibit the NF-κB signaling pathway by binding to MyD88 and removing K63-linked ubiquitin chains from MyD88^27^. Our mapping assays revealed that both BCA2 and OTUD4 bind to the same region of MyD88 (Fig. 4F and Fig. 5A). We speculated that BCA2 prevents the binding of OTUD4 to MyD88. Indeed, endogenous immunoprecipitation experiments following BCA2 knockout showed an increased interaction between MyD88 and OTUD4 (Fig. 5B). In agreement with this result, overexpression of BCA2 resulted in a decreased interaction between BCA2 and OTUD4 (Fig. 5C). Consistently, BCA2 knockout decreased the level of MyD88 ubiquitination in HCC1806 cells (Fig. 5D). It is well known that recruitment of IRAKs by MyD88 is crucial for the activation of the NF-κB pathway 28. Accordingly, deletion of BCA2 impairs LPS-induced recruitment of IRAK1 to MyD88 (Fig. 5E and Fig. S4A). These findings suggested that BCA2 prevents the binding of OTUD4 to MyD88, which in turn, increases the ubiquitination of MyD88, independent of its E3 ligase enzymatic activity.

### Association between BCA2 Expression and Breast Cancer Prognosis

**Figure 6:**
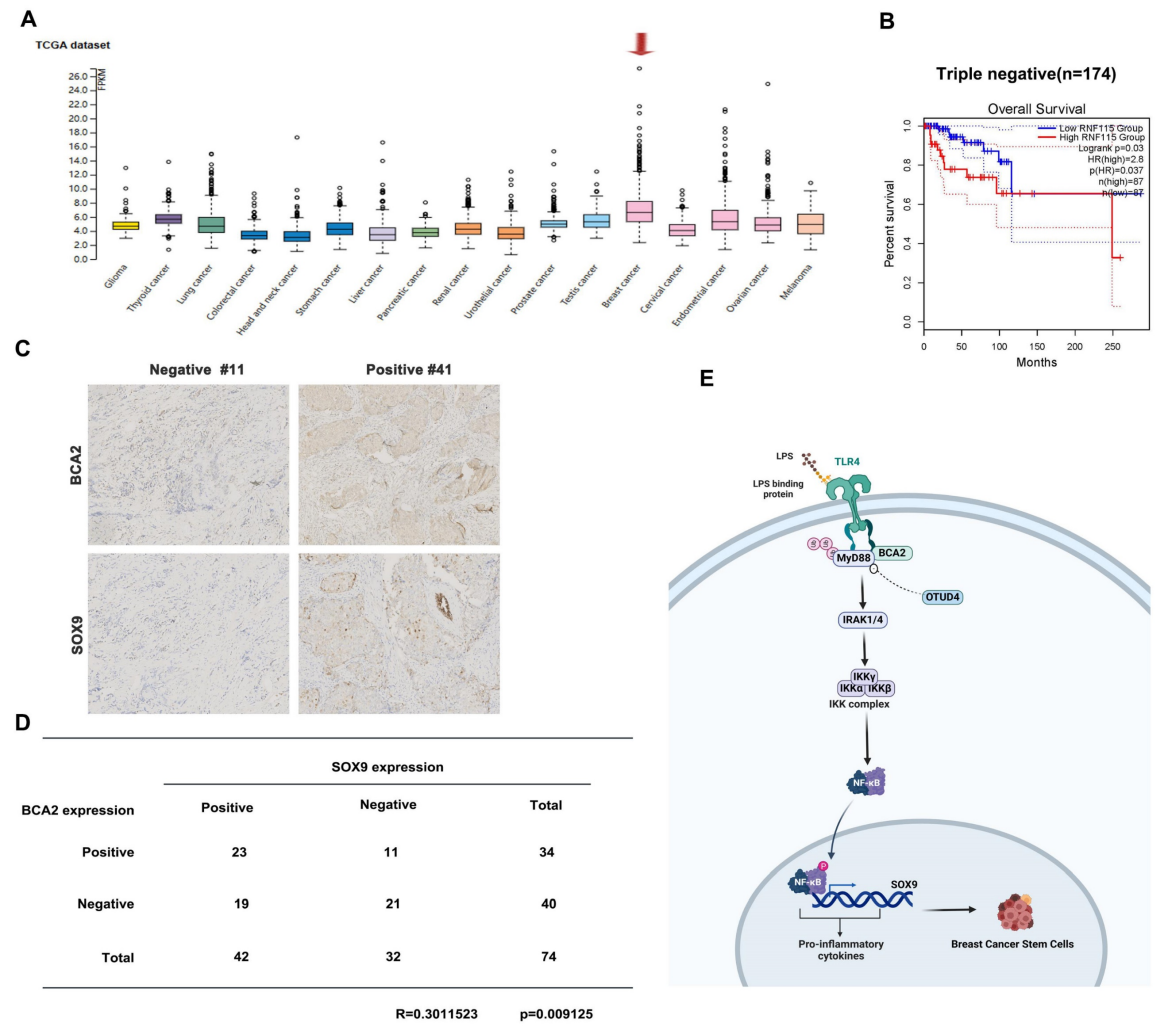
Association of BCA2 expression with breast cancer prognosis. **(A)** The HPA database was used to evaluate the expression of BCA2 in tumors. **(B)** Overall survival of patients with high (>35%, red curve) and low (<65%, blue curve) BCA2 levels analyzed by GEPIA using the log-rank test. **(C)** The expression levels of SOX9 and BCA2 were positively correlated in breast cancer patients. **(D)** Representative IHC results for SOX9 and BCA2 are shown. **(E)** Schematic representation of the role of BCA2 in the regulation of BCSCs.

To investigate the relative clinical information of BCA2 in TNBC, we investigated the correlation between BCA2 expression and the prognosis of breast cancer patients by analyzing pancancer data in the HPA (The Human Protein Atlas (v20.proteinatlas.org)) and GEPIA databases (Gene Expression Profiling Interactive Analysis (cancer-pku.cn)). We found that BCA2 is highly expressed in breast cancer (Fig. 6A). Moreover, there was a significant association between elevated BCA2 expression and poorer prognosis in TNBC patients (HR=2.8, P=0.037) (Fig. 6B). To test whether BCA2 and SOX9 are co-expressed in TNBC, we examined the protein levels of BCA2 and SOX9 in clinical samples from tissue microarrays of 74 TNBC patients by immunohistochemistry (IHC). We observed a positive correlation between BCA2 and SOX9 expression in these samples (R=0.3010523, P=0.009125) (Fig. 6C,D). Taken together, BCA2 promotes breast cancer stemness by activating LPS-mediated NF-κB signaling and upregulating the expression of SOX9 (Fig. 6E).

## Discussion

CSCs have the ability to self-renew and differentiate and play a central role in driving cancer initiation and progression. CSCs have emerged as promising therapeutic target cells 29. TNBC has an increased propensity for early metastasis and lower five-year survival rates than non-TNBC 30. In addition, studies have shown that TNBC has a more abundant population of stem cells characterized by the presence of CD44^high^/CD24^low^ and ALDH1+ compared to luminal and HER2 subtypes 31, 32. Therefore, elucidating the molecular mechanisms of CSCs in TNBC is crucial to identify new therapeutic targets and improve breast cancer therapy. Our study demonstrated that BCA2 promotes the proportion of BCSCs in TNBC.

The role of BCA2 in cancer has received limited attention, with studies focusing primarily on breast, lung, and gastric cancers. According to recent studies, BCA2 promotes survival in lung adenocarcinoma cells by promoting Wnt/β-catenin signaling pathway activation through APC ubiquitination. In addition, BCA2 promotes the progression of lung adenocarcinoma through targeting p53 ubiquitination 33, 34. Conversely, a different study reported that BCA2 induces the ubiquitination of c-Myc, thereby inhibiting lung cancer cell growth 35. In gastric cancer, BCA2 interacts with STX17 to induce autophagosome maturation, eventually promoting gastric cancer progression 22. Notably, BCA2 expression is elevated in breast cancer cells and tissues and is inversely associated with lymph node metastasis and disease-free survival following local recurrence 25. Based on our previous investigations, BCA2 causes the degradation of p21 by ubiquitination, leading to breast cancer cell proliferation 19. In addition, several studies have highlighted the involvement of BCA2 in promoting migration and enhancing DNA damage repair in breast cancer 18, 36. However, Zhang et al. found that BCA2 negatively regulates Toll-like receptor (TLR)-mediated autoimmunity and antimicrobial immunity and that overexpression of BCA2 inhibits the postendoplasmic reticulum trafficking of TLRs and their mediated signaling pathways 21, 24. However, our results show that BCA2 promotes breast cancer progression and acts by promoting TLR4-mediated NF-κB activation. Our research has provided the first evidence regarding the critical role of BCA2 in promoting the proportion of BCSCs in TNBC.

Interactions between the immune system and tumors are dynamic in nature. TLRs are a widely studied group of pattern recognition receptors; among them, TLR4 has been extensively studied and is known to be involved in the development of several types of tumors, such as breast cancer. TLR4 signaling includes both MyD88-dependent and MyD88-independent pathways 37. LPS, a component present in the outer membrane of gram-negative bacteria, is a well-known TLR4 activator. In the presence of lipopolysaccharide binding protein (LBP), CD14 binds to LPS and plays a critical role in loading LPS into the TLR4/MD2 complex (MD2, also known as LY96). Subsequent activation of the TLR4/MD2 complex initiates downstream signaling cascades, including NF-κB signaling activation, leading to the production of proinflammatory cytokines 38. Notably, the expression levels of TLR4 and its downstream adaptor protein MyD88 are significantly elevated in breast cancer compared to adjacent normal tissue, and the upregulation is associated with a poor prognosis 39. LPS systemically induces migratory, invasive, and angiogenic properties in breast cancer cells. Intraperitoneal injection of LPS in BALB/c mice with breast cancer has been reported to induce angiogenesis 40. TLR4 activation promotes the secretion of growth-promoting factors, which regulate the proliferation of cancer cells in p53-mutant breast cancer 41. Notably, our findings highlight the substantial involvement of BCA2 in the TLR4-mediated MyD88-dependent signaling pathway. LPS was reported to promote the growth of microspheres, indicating that these progenitor cells can sense and respond to microbial products 42. Based on our results, we propose that BCA2 promotes the proportion of BCSCs in TNBC through the LPS-mediated MyD88-dependent NF-κB signaling pathway.

The SOX9 protein serves as an oncogene, exerting its influence on initiation, proliferation, migration, chemoresistance and stem cell maintenance pathways 43. Its expression is notably elevated in breast cancer tissues compared to normal tissues, particularly in ERα-negative breast cancers, and its high expression indicates shorter overall survival. SOX9 plays a crucial role in breast cancer progression and the preservation of stem cells. Several studies have revealed that SOX9 interacts with calcineurin, specifically CDH4 and CDH17, thereby maintaining the stem cell phenotype in TNBC 44. In this study, we showed that SOX9, as a significant downstream target of BCA2 upon LPS stimulation, promotes the proportion of BCSCs in TNBC. SOX9 cooperates with the transcription factor Slug to determine mammary stem cell (MaSC) status, and coexpression of both Slug and SOX9 promotes the development of breast cancer, as well as the ability for metastasis 45.

Numerous studies have demonstrated the involvement of NF-κB in breast cancer tumorigenesis and resistance to endocrine therapy 46, 47. Our study found that LPS-induced NF-κB could promote the proportion of BCSCs in the presence of BCA2. Consequently, targeting BCA2 holds promise as a therapeutic approach capable of counteracting the effects mediated by LPS. In conclusion, BCA2 promotes the activation of the NF-κB signaling pathway by LPS in TNBC. The specific mechanism is that BCA2 interacts with and promotes the binding of TLR4 and MyD88. In addition, BCA2 prevents OTUD4, a deubiquitinating enzyme, from binding to MyD88 and removing K63 polyubiquitin chains, thereby promoting the NF-κB signaling pathway. Our findings shed light on the critical role of BCA2 in promoting the proportion of BCSCs in TNBC and provide potential therapeutic targets for the treatment of breast cancer, particularly in targeting BCSCs.

## Supplementary Material

Supplementary figures.
