# Supplementary material for: E3 ubiquitin ligase BCA2 promotes breast cancer stemness by up-regulation of SOX9 by LPS
Source: Int J Biol Sci. 2024 Apr 29;20(7):2686–97. doi: 10.7150/ijbs.92338 (PMC11077363; doi:10.7150/ijbs.92338)
Supplement: Supplementary file 1 — Supplementary figures. [file ijbsv20p2686s1.pdf]

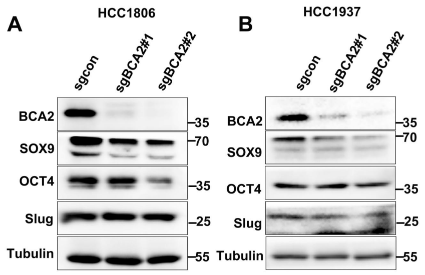

Supplementary Figure 1

A) Immunoblot analysis of SOX9, OCT4, Slug and tubulin after knockout of BCA2 in HCC1806 cells.

B) Immunoblot analysis of SOX9, OCT4, Slug and tubulin after knockout of BCA2 in HCC1937 cells.

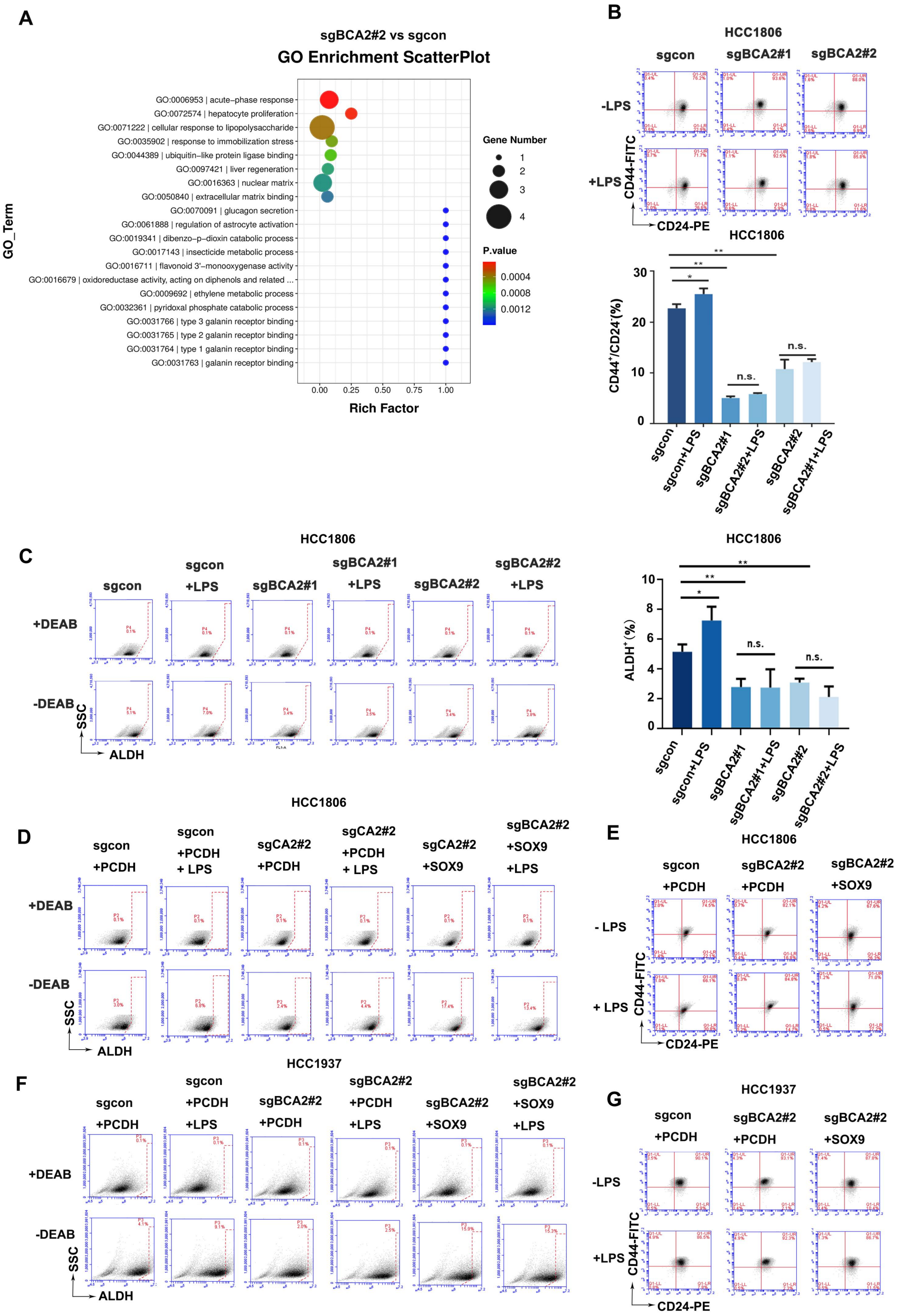

Supplementary Figure 2

A) GO showing enrichment of cellular response to LPS in BCA2 differentially expressed genes based on RNA-Seq data (sgBCA2#2 vs sgcon).

B) $CD44^+/CD24^-$ BCSC population analysis in HCC1806 cells with BCA2 knockout followed by stimulation with LPS (50 ng/mL). Statistical results of the $CD44^+/CD24^-$ BCSC population in HCC1806 cells (*, $p < 0.05$ **, $p < 0.01$, n.s. , not significant).

C) ALDH activity detected by ALDEFLUOR assay in HCC1806 cells with BCA2 knockout followed by stimulation with LPS (50 ng/mL). Statistical results of the ALDH+ BCSC population in HCC1806 cells (*, $p < 0.05$ **, $p < 0.01$, n.s. , not significant).

D) ALDH activity detected by ALDEFLUOR assay in HCC1806 cells with BCA2 knockout followed by stimulation with LPS (50 ng/mL) or BCA2 knockout followed by SOX9 rescue and stimulation with LPS (50 ng/mL).

E) $CD44^+/CD24^-$ BCSC population analysis in HCC1806 cells with BCA2 knockout followed by stimulation with LPS (50 ng/mL) or BCA2 knockout followed by SOX9 rescue and stimulation with LPS (50 ng/mL).

F) ALDH activity detected by ALDEFLUOR assay in HCC1937 cells with BCA2 knockout followed by stimulation with LPS (50 ng/mL) or BCA2 knockout followed by SOX9 rescue and stimulation with LPS (50 ng/mL).

G) $CD44^+/CD24^-$ BCSC population analysis in HCC1937 cells with BCA2 knockout followed by stimulation with LPS (50 ng/mL) or BCA2 knockout followed by SOX9 rescue and stimulation with LPS (50 ng/mL).

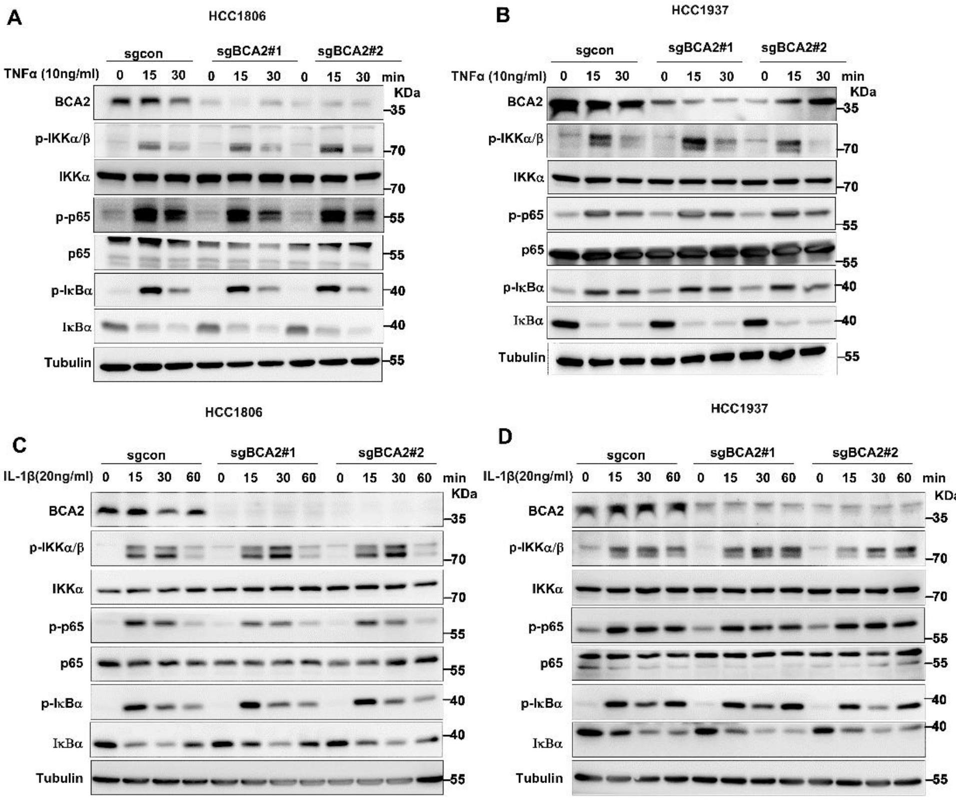

Supplementary Figure 3

A) Immunoblot analysis of total and phosphorylated IKKα/β, p65, and IκBα in BCA2 knockout and control HCC1806 cells stimulated with TNFα (10 ng/mL) for 0-30 minute.

B) Immunoblot analysis of total and phosphorylated IKKα/β, p65, and IκBα in BCA2 knockout and control HCC1937 cells stimulated with TNFα (10 ng/mL) for 0-30 minutes.

C) Immunoblot analysis of total and phosphorylated IKKα/β, p65, and IκBα in BCA2 knockout and control HCC1806 cells stimulated with IL-1β (20 ng/mL) for 0-60 minutes.

D) Immunoblot analysis of total and phosphorylated IKKα/β, p65, and IκBα in BCA2 knockout and control HCC1937 cells stimulated with IL-1β (20 ng/mL) for

0-60 minutes.

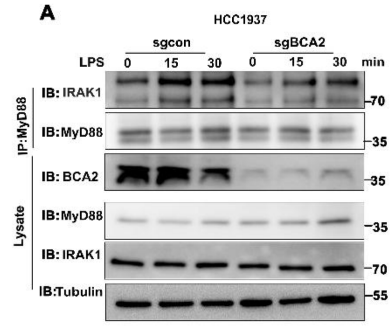

Supplementary Figure 4

A) HCC1937 cells with BCA2 knockout were stimulated with LPS (500 ng/ml) for 0-30 minutes before immunoprecipitation and immunoblot analysis.
